# Resurfacing hip arthroplasty better preserves a normal gait pattern at increasing walking speeds compared to total hip arthroplasty

**DOI:** 10.1080/17453674.2019.1594096

**Published:** 2019-04-01

**Authors:** Davey M J M Gerhardt, Thijs G Ter Mors, Gerjon Hannink, Job L C Van Susante

**Affiliations:** a Department of Orthopedics, Rijnstate Hospital, Arnhem;;; b Department of Operating Rooms, Radboud University Medical Center, Nijmegen, The Netherlands

## Abstract

Background and purpose — Gait analysis performed under increased physical demand may detect differences in gait between total (THA) versus resurfacing hip arthroplasty (RHA), which are not measured at normal walking speed. We hypothesized that patients after RHA would reach higher walking speeds and inclines compared with THA. Additionally, an RHA would enable a more natural gait when comparing the operated with the healthy contralateral hip.

Patients and methods — From a randomized controlled trial comparing THA with RHA with at least 5 years’ follow-up patients with a UCLA score of more than 3 points (n = 34) were included for an instrumented treadmill gait analysis. 25 patients with a unilateral implant (primary analysis—16 THA versus 9 RHA) and 9 patients with a bilateral implant (sub-analysis—n = 5 RHA + THA; n = 4 THA + THA). Spatiotemporal parameters, ground reaction forces, and range of motion were recorded at increasing walking speeds and inclines. Functional outcome scores were obtained.

Results — At a normal walking speed of 1.1 m/s and at increasing inclines no differences were recorded in gait between the 2 groups with a unilateral hip implant. With increasing walking speed the RHA group reached a higher top walking speed (TWS) (adjusted difference 0.07 m/s, 95% CI –0.11 to 0.25) compared with THA. Additionally, RHA patients tolerated more weight on the operated side at TWS (155 N, CI 49–261) and as such weight-bearing approached the unaffected contralateral side. For the RHA group a “between leg difference” of 8 N (CI 3–245) was measured versus –129 N (CI –138 to –29) for THA (adjusted difference 144 N, CI 20–261). Hip flexion of the operated side at TWS was higher after RHA compared with THA (adjusted difference 8°, CI 1.7–14).

Interpretation — In this study RHA patients reached a higher walking speed, and preserved a more normal weight acceptance and a greater range of hip flexion against their contralateral healthy leg as compared with patients with a THA.

Increasing numbers of young patients choose hip arthroplasty instead of accepting hip impairment. In an attempt to increase implant durability and future revision options the metal on metal (MoM) resurfacing hip arthroplasty (RHA) was introduced, improving implant stability with the use of larger femoral head diameters and preservation of femoral bone stock (Amstutz et al. 2004, Grigoris et al. 2006, Gerhardt et al. 2015). Patients benefit from regaining hip function near to normal as gait analysis studies and questionnaires have shown (Mont et al. 2007, Bisseling et al. 2015). However, the use of RHA has decreased over the past decade due to concerns about adverse reactions to metal debris (Langton et al. 2010). Still, the hip resurfacing concept, restoring patients’ mobility particularly in young active patients, remains relevant since previous studies have reported somewhat better functional outcome after RHA versus THA (Pollard et al. 2006, Heilpern et al. 2008, Haddad et al. 2015). So far, only 2 randomized controlled trials have been performed comparing postoperative gait between RHA and THA (Lavigne et al. 2010, Petersen et al. 2011). In these studies, the clinically perceived benefit of RHA compared with conventional THA on patient mobility and gait could not be confirmed. However, these studies may not be entirely conclusive since a limited number of patients were enrolled and measurements were done at normal walking speed. More modern gait analysis does allow assessment of patients’ gait pattern at increasing walking speeds and inclines. The advantage of using an instrumented treadmill is the ability to continuously increase speed and walking incline to detect gait differences that may not be detected at a normal or slow walking speed.

In this study a modern instrumented treadmill assisted gait analysis after RHA versus THA was performed where spatiotemporal, kinematic, and kinetic data could be continuously monitored under increasing walking speed and incline. We hypothesized that in this way RHA patients would still prove to preserve a more normal gait pattern of the operated leg similar to the gait pattern of the healthy contralateral leg.

## Patient and methods

**Figure 1.**
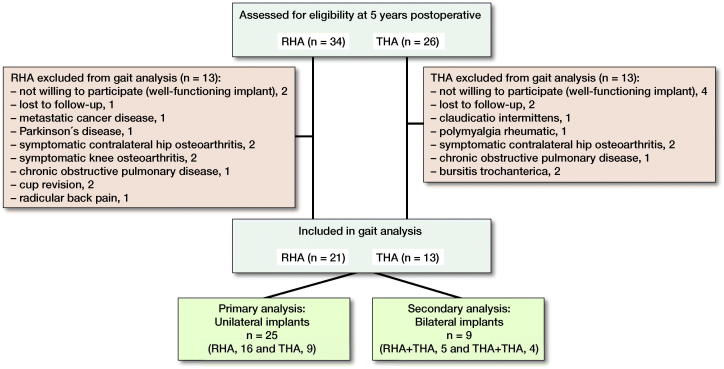
Flowchart of study.

The study group included RHA and THA patients from a larger randomized controlled trial to compare RHA against a conventional small-diameter MoM THA with at least a complete 5-year follow up (Smolders et al. 2010). 34 RHA and 26 THA patients were available to participate in this instrumented gait analysis follow-up study. Only relatively active patients, who did not use walking aids during daily living, with more than 3 points according to the university of California at Los Angeles (UCLA) activity score at 5 years’ follow-up were approached. Exclusion criteria were contralateral hip osteoarthritis, presence of a total knee arthroplasty, or any musculoskeletal disorder affecting patients’ gait other than the hip implant. 34 patients could be included for gait analysis. Patients were categorized into 2 groups: (1) 25 patients with a unilateral hip implant (16 RHA and 9 THA), and (2) 9 patients with a bilateral hip implant (4 THA + THA and 5 RHA + THA). The primary analysis concerns patients with a unilateral hip implant, comparing THA with RHA; the secondary analysis concerns patients with bilateral implants. (Figure 1, Table 1).

**Table 1.** Clinical details of the 2 groups of patients with a unilateral hip implant

Factor	RHA (n = 16)	THA (n = 9)	p-value
Sex (women/men)	5/11	1/8	0.4^a^
Mean body mass index (SD)	26 (3)	28 (5)	0.2^b^
Length, cm (SD)	177 (13)	180 (9)	0.5^b^
Weight, kg (SD)	82 (19)	91 (21)	0.3^b^
Mean age at surgery (SD)	52 (10)	57 (8)	0.2^b^
Mean follow-up in years (SD)	6.3 (1)	6.2 (0)	0.9^b^

a Fisher’s exact probability test

b Student’s t-test.

### Functional questionnaires

2 weeks prior to the gait analysis all patients completed the Western Ontario and McMaster Universities Osteoarthritis Index (WOMAC), EuroQol 5D (EQ-5D), UCLA activity score, and the Oxford Hip Score (OHS) to establish patient-reported outcome after surgery (Table 2).

**Table 2.** Clinical scores according to the UCLA activity score, Oxford Hip Score (OHS: best–worst 12–60 points scoring), EQ-5D visual analogue scale, and WOMAC hip score (best to worst 0–94 points scoring). Values are mean (SD)

Unilateral	RHA (n = 16)	THA (n = 9)	p-value
OHS	14 (3)	14 (2)	0.9
UCLA	6.9 (2.4)	7.3 (2.4)	0.7
EQ-5D VAS	80 (7)	78 (10)	0.6
WOMAC	4 (5)	4 (5)	0.8

Student’s t-test was performed.

### Gait analysis

**Figure 2.**
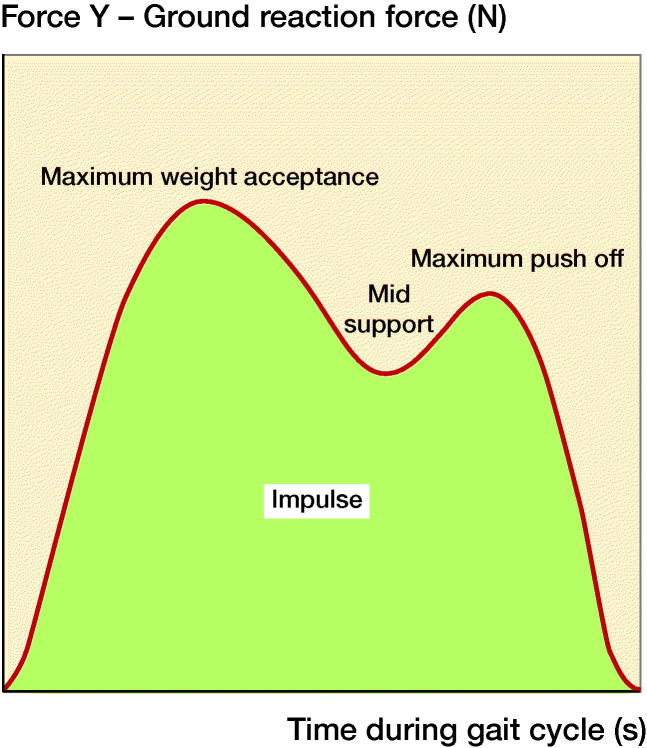
Illustration of the ground reaction force (N) plotted against time (s) during a gait cycle from heel-strike to toe-off, with the maximum weight acceptance (first peak), mid support, maximum push (second peak) off, and the impulse (area under the curve).

Patients were assessed by a Gait Real-time Analysis Interactive Lab (GRAIL; Motek Medical, Amsterdam, the Netherlands). A 3D motion capture system with an instrumented dual-belt treadmill was employed, with force plates underneath both belts to record the kinetics of each step, left and right, independently at increasing speed and inclination (maximum 10 degrees). A motion-capture system with 24 anatomic placed body markers on the lower extremities, pelvis, and spine is integrated in the GRAIL to record changes in body position and range of motion of the hip joint during each gait cycle. Since body markers were placed on anatomical landmarks including the lateral femoral epicondyle, greater trochanter of the femur, anterior and posterior superior iliac spine, sacral bone, and Th10, hip flexion and extension could be monitored after correction for concomitant spine motion. The 3D marker trajectories were collected (100 Hz) with a 10-camera 3D motion capture system (Vicon Nexus, Oxford Metrics Ltd, Oxford, UK) and processed in D-flow (Motekforce Link, Amsterdam, the Netherlands). Kinetic data were collected from the force plates (Forcelink, 12 channels, sample frequency 1000 Hz) during the stance phase according to the gait analysis protocol published by Aqil et al. (2013) resulting in 4 variables for analysis: maximum weight acceptance, mid support, maximum push off, and impulse. Maximum weight acceptance and maximum push off are the first and second force peaks in the stance phase with the mid support force being the lowest point between both peaks. The impulse is defined as the total force throughout the stance phase or the area under the curve (Figure 2). Kinetic data obtained from the force plates was normalized for bodyweight towards a standard 80 kg.

With the motion capture system the spatiotemporal parameters speed, step length, stride time, and cadence, as well as the kinematic data of hip range of motion, were continuously recorded. All patients were tested by 2 independent physical therapists, blinded for the implant type and side. Testing followed a 6-minute acclimatization period at a fixed 1.11 m/s (4 km/h) speed to eliminate inconsistencies in the patients’ gait due to lack of warming up (Matsas et al. 2000, Wiik et al. 2012). Patients walked wearing shoes, without any support or walking aids on the treadmill.

Flat ground walking started at 1.11 m/s and was increased by 0.28 m/s (1 km/h) every 20 s up to the patient’s top walking speed (TWS). After a 5-minute break, patients were asked to walk uphill at a fixed speed of 1.11 m/s, with increments of 1° every 20 s. The treadmill was inclined up to the patient’s maximum walking incline (TWI), with a maximum treadmill incline of 10°, which corresponds to intensive hiking.

### Statistics

All data extracted from the GRAIL system were analyzed using MATLAB (R2014b, The MathWorks Inc, Natick, MA, USA). Force calculations were performed only on correctly measured steps. A correctly measured step was defined as a deviation from the mean of all steps of less than 2 times the standard deviation.

Descriptive statistics were used to summarize the data. Independent samples t-tests and non-parametric independent t-tests were used to assess differences between baseline scores. As the participants were selected from a previously performed randomized controlled trial (Smolders et al. 2010), no prior power calculation was performed. Linear regression was used to test between-group differences (RHA vs. THA) in spatiotemporal, kinetic, and kinematic parameters while adjusting for sex, age at surgery, BMI, and UCLA score. Results are presented as means with 95% confidence intervals (CI). Additional analyses were performed on the 9 patients with bilateral hip implants; an RHA combined with a THA (n = 5), or a bilateral THA (n = 4). In these small groups, paired t-tests were used to assess the between-leg differences regarding the ground reaction forces in both groups, with kinematic and spatiotemporal parameters. Differences were considered statistically significant with a p-value < 0.05. All statistical analyses were performed using R version 3.5.1 (R Foundation for Statistical Computing, Vienna, Austria).

### Ethics, registration, funding, and potential conflicts of interest

Approval from the regional ethics committee was obtained for the gait analysis (LTC 2015-0576/METC NL50830.091.14).Written informed consent was obtained from all patients. The study was registered at ClinTrials.gov (NCT02484781). An independent institutional research grant was obtained (Rijnstate Vriendenfonds). There are no conflicts of interest to be reported by any of the authors.

## Results

### Primary analysis

#### Clinical outcome

Patient characteristics and surgical data for the primary analysis are summarized in Table 1. 16 unilateral RHA and 9 unilateral THA were included. Clinical outcome scores are presented in Table 2. Overall both groups presented with a good OHS, UCLA score, EQ-5D VAS, and WOMAC without clinically relevant differences.

### Primary gait analysis (patients with a unilateral hip implant)

1. *Fixed speed (1.11 m/s), no incline (0°).* No statistically significant differences were measured between the RHA and the THA regarding the spatiotemporal, kinetic, and kinematic parameters recorded when walking at a normal speed of 1.11 m/s (4 km/h) (Tables 3–5, see Supplementary data). In addition, no statistically significant discrepancy between the implanted hip and the contralateral hip was observed irrespective of the type of implant.
2. *Increasing walking speed towards TWS with no incline (0°).* The RHA group reached a higher TWS than the THA group, 2.03 m/s (SD 0.2) versus 1.92 m/s (0.2), respectively (adjusted difference 0.07 m/s, CI –0.11 to 0.25). No differences were seen regarding the stride time, stride length, or cadence, both parameters showed similar adaptation towards TWS (Table 3, see Supplementary data). For the THA group an increasing walking speed coincided with an increasing discrepancy in weight acceptance between the implanted (977 N (103)) and the contralateral healthy hip (1,106 N (85)) (Δ = –129 N at TWS (CI –138 to –29). As for the RHA group a minor between-legs difference was measured of 1,132 N (153) versus 1,124 N (114) for the contralateral side (Δ = 8 N at TWS (CI 3–245). Thus the adjusted difference in weight acceptance between legs at TWS for the RHA group and the THA group was 144 N (CI 20–261). For the other ground reaction forces recorded, no statistically significant differences were seen at TWS (Table 4a, see Supplementary data). Regarding hip range of motion at an increasing walking speed up to patients’ TWS, hip flexion increased from 35° (6) towards 47° (5) after RHA versus 33° (8) towards 39° (6) after THA. Hip flexion of the operated leg at TWS was higher after RHA (adjusted difference 8° (CI 1.7–14). Additionally, a minor between leg difference was measured (adjusted difference 2.8, CI –7.4 to 1.8) (Table 5, see supplementary data).
3. *Fixed walking speed and an increasing incline towards TWI.* At an increasing incline with a fixed normal walking speed (1.11 m/s) no statistically significant differences were seen between RHA and THA at patients’ TWI (Table 3, see Supplementary data). Regarding the ground reaction forces measured, no differences between groups or between the operated and the contralateral hip were measured at increasing inclines (Table 4a, see Supplementary data). No difference was measured in patients’ hip range of motion between legs or between groups for hip flexion and extension (Table 5, see Supplementary data).

### Secondary gait analysis: patients with bilateral hip implants

The group of 5 bilateral patients (4 women) with an RHA on one side and a THA on the other side had a mean BMI of 28 (4), mean UCLA of 7.8 (0.8) and a mean age at surgery of 60 (4) years. From the small number of patients, obviously no statistically significant differences could be detected in between-leg differences regarding the ground reaction forces, stride length, stride time, and hip range of motion. Overall, at normal walking speed and TWI all mean values were comparable between the two legs (Table 4b and 6, see Supplementary data). This also accounted for TWS, except that the difference in maximum weight acceptance between the two legs increased in favor of the side with an RHA (adjusted difference 45 N (CI –63 to 153).

The group of 4 patients (3 women) with a bilateral THA had a mean BMI of 24 (2), mean UCLA of 7.4 (1), and mean age at surgery of 54 years (12). Again, overall no between-leg differences were measured at normal walking speed, TWS, or TWI regarding the ground reaction forces, stride length, stride time, and hip range of motion (Table 4b and 6, see Supplementary data). This time the difference in weight acceptance between the two legs (THA and THA) was less (2 N, CI –82 to 87).  

## Discussion

Postoperative gait differences between RHA and THA remain controversial regarding presumed benefits for RHA. In this treadmill-assisted gait analysis indeed no differences in gait pattern were measured at a fixed flat walking speed of 1.11 m/s, nor at increasing inclines with a fixed flat walking speed. However, with increasing speeds towards patients’ TWS, patients with an RHA had a weight acceptance on the operated hip similar to the weight acceptance of the healthy contralateral hip. In the group of patients with a THA this weight acceptance on the operated leg was relatively lower, resulting in a higher between-leg difference at top walking speed. In addition, a greater range of motion in the hip joint was measured and a trend towards a higher top walking speed and a greater stride length was observed after RHA. The primary gait analyses focused on patients with a unilateral hip implant. In an attempt to maximize the potential inclusion of patients available for gait analysis a secondary gait analysis was also performed on patients with bilateral hip implants. The 2 secondary gait analyses of patients with a bilateral hip implants (RHA + THA and bilateral THA) confirmed overall the outcome of the primary analyses without clear between-leg differences in the evaluated outcome parameters, in particular for the bilateral THA group. Interestingly, patients with an RHA and a THA revealed a similar weight acceptance between legs at normal walking speed whereas at TWS the difference in weight acceptance increased in favor of the RHA side. With the small sample size this difference was statistically not significant.

2 randomized controlled trials on gait analysis have already reported on similar postoperative walking speed and gait restoration after RHA versus THA without a statistically significant difference between groups (Lavigne et al. 2010, Petersen et al. 2011). However, in contrast to our study Petersen et al. (2011) assessed gait adaptation at patients’ comfortable walking speed at 6 and 12 weeks after surgery in 30 patients (15 RHA versus 15 THA). A respective non-significant difference in speed increase from 6 to 12 weeks after surgery was observed of 1.19 (0.3) to 1.32 (0.2) m/s (RHA) versus 1.10 m/s (0.3) to 1.25 m/s (0.2) (THA). No differences in kinematic and kinetic parameters were measured between groups at these comfortable walking speeds. Lavigne et al. (2010) reported on a non-significant difference in comfortable walking speeds favoring RHA (n = 24) at 12 months postoperatively. In that study, however, RHA was compared with a large diameter head THA (n = 15) instead of a more conventional small diameter head THA. Both these earlier studies differ importantly from our study as most parameters were evaluated only at normal, comfortable walking speeds and weight acceptance of the operated leg was not assessed. For this reason we feel that these earlier studies missed the encountered differences in our study. A more recent non-randomized study by Wiik et al. (2012) also used gait analysis on an instrumented treadmill to compare 22 RHA, 22 THA, and a control group (n = 23). They reported on a top walking speed (TWS) of 2.06 m/s (0.22) after RHA versus 1.90 m/s (0.19) after THA (p < 0.05), whereas the control group without a hip arthroplasty reached 2.08 m/s (SD 0.17). As such that study also confirmed a better performance for RHA with increasing walking speeds; however, it may have been biased since cohorts were selected. The strength of our study is that these findings were also confirmed in patients from a randomized study.

The use of a large femoral head diameter in RHA and the absence of an intramedullary stem in the femur may explain the perceived more natural gait frequently claimed by patients after RHA. Our findings objectively support this assumption as indicated by a more natural postoperative gait restoration seen after RHA compared with conventional THA (Figure 3 shows typical examples of gait adaptation after RHA and THA). Besides the larger range of motion that can be obtained with a larger femoral head diameter (Burroughs et al. 2005), in particular the absence of an intramedullary stem did allow for weight acceptance on the operated leg comparable to the non-operated contralateral side. Earlier studies already recognized this tendency towards a more physiological gait after RHA (Daniel et al. 2004, Mont et al. 2007, Aqil et al. 2013). For example, Aqil et al. (2013) performed an instrumented treadmill-assisted gait analysis in 9 patients with bilateral hip arthroplasties, THA (head diameters range from 28 to 38mm) on one side versus RHA contralaterally. A strong correlation between increasing speeds and increasing between-leg differences in ground reaction force was also described.

Figure 3.(a) Typical example of gait adaptation towards top walking speed (TWS) after RHA. Similar leg differences are seen at 4 km/h and at TWS (in this case at 8 km/h) between the healthy and operated leg.

**Figure:**
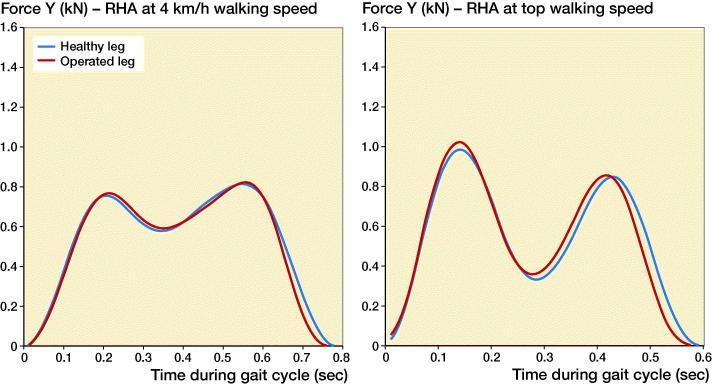

**Figure:**
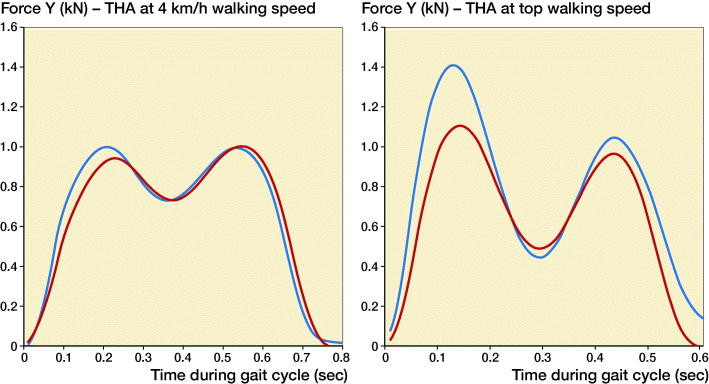

The presence of an intramedullary stem obviously stiffens the femur, which in turn decreases weight tolerance of that femur. The fact that with RHA such a stemmed device can be avoided appears to be a benefit. It should be noted, however, that only with gait analysis using increasing walking speeds could this benefit for RHA be quantified and as such it may not be as clinically significant. In addition, this benefit for RHA has to be balanced against the increasing concern around metal-on-metal articulation (Smith et al. 2012, Bolognesi and Ledford 2015). For the future it may remain interesting to focus on research allowing for the resurfacing concept whilst avoiding metal-on-metal articulations (Van Susante et al. 2018).

An important strength of this study is that patients were recruited from a randomized trial and that selection bias could be avoided, which may be a risk when cohort series are compared. In addition the gait analysis was performed on an advanced instrumented treadmill with the use of motion capture, which ensured computerized measurements and provided a simultaneous registration of spatiotemporal, kinetic, and kinematic measurements.

Some potential limitations also have to be discussed. We decided to perform an additional gait analysis on patients already included in an RCT with 5 years’ follow-up completed. Thus the available number of patients was predetermined and could not be increased to improve the power of the study. In addition, patient inclusion was based on the UCLA score and the patient’s comorbidities recorded in their medical file that could have influenced the gait analysis. This might have induced selection bias and introduced imbalances in covariates, although patient demographics and functional scores were similar. However, in our analyses we adjusted for these imbalances. Moreover, in a secondary analysis, bilateral cases were analyzed and confirmed our findings. Besides, due to the selection criteria a rather homogeneous study group of relatively active individuals was established, which may also have strengthened the study potential to identify an implant-related difference in this rather small number of patients.

For the statistical analysis of the ground reaction forces patient bodyweight was normalized to 80 kg. Since the main focus of this study was detecting potential inter-patient (between leg) and not intra-patient differences this correction for bodyweight did not bias the results. Finally, since the number of available patients was predetermined no prior power calculation for this study was performed.

In summary this study confirms that at a normal walking speed (1.11 m/s) no major differences in patient postoperative gait pattern can be expected comparing RHA with conventional THA. However, with increasing walking speeds RHA patients preserved a more normal weight acceptance and a greater range of hip flexion against their contralateral healthy leg as compared with patients with a THA. We believe that maintenance of a large femoral head diameter and avoidance of stiffening the femur with an intramedullary stem are the main contributors to this benefit for RHA. Obviously, the concerns around adverse reaction to metal debris from a metal-on-metal articulation so far remain an important disadvantage for RHA and should be balanced against this benefit in gait; future innovations avoiding metal-on-metal articulation in resurfacing remain interesting.  

### Supplementary data

Tables 3–6 are available as supplementary data in the online version of this article, http://dx.doi.org/10.1080/17453674. 2019.1594096  

The study was designed by DG and JLCvS. DG and TM carried out the data processing. The quality control of data was performed by DG, TM, and GH. DG and GH performed the statistical analysis. All authors interpreted the results. The manuscript preparation and editing were done by DG. All the authors reviewed the final manuscript.

*Acta* thanks Håkan Hedlund and Marketta Henriksson for help with peer review of this study.

(b) Typical example of gait adaptation towards top walking speed (TWS) after THA. Minor between leg differences are measured at 4 km/h between the healthy and operated leg; however, at TWS this between-leg difference increases, in this case at 7 km/h.

## Supplementary data

## Supplementary Material

Supplementary Material
