# Supplementary material for: Resurfacing hip arthroplasty better preserves a normal gait pattern at increasing walking speeds compared to total hip arthroplasty
Source: Acta Orthop. 2019 Apr 1;90(3):231–6. doi: 10.1080/17453674.2019.1594096 (PMC6534262; doi:10.1080/17453674.2019.1594096)
Supplement: Supplementary Material [file IORT_A_1594096_SM0244.pdf]

Supplementary data

Table 3. Spatiotemporal parameters regarding patients with a unilateral hip implant: flat walking at 1.11 m/s (4 km/h), patients' top walking speed (TWS), and top walking incline (TWI) (maximum treadmill incline 10°). Values are mean (SD) or adjusted between-group differences with CI (aD)

	Speed (m/s)		Mean stride length (m)		Mean stride time (s)		Cadence (steps/min)	
	RHA	THA	RHA	THA	RHA	THA	RHA	THA
Walking 1.11 m/s	1.11	1.11	1.25 (0.07)	1.25 (0.06)	1.12 (0.06)	1.12 (0.06)	53.6 (2.9)	53.5 (3.0)
aD	–		0.00 (–0.00 to 0.00)		0.00 (–0.00 to 0.00)		–0.02 (–0.07 to 0.04)	
TWS	2.03 (0.21)	1.92 (0.22)	1.75 (0.18)	1.64 (0.18)	0.87 (0.05)	0.85 (0.03)	69.5 (3.9)	70.5 (2.8)
aD	0.07 (–0.11 to 0.25)		0.00 (–0.02 to 0.01)		0.00 (–0.01 to 0.00)		–0.17 (–0.27 to 0.61)	
TWI	9.50 (1.41)	9.22 (1.39)	1.23 (0.07)	1.22 (0.09)	1.11 (0.07)	1.10 (0.08	54.4 (3.3)	54.6 (3.8)
aD	0.25 (–0.93 to 1.43)		0.00 (–0.00 to 0.00)		0.00 (–0.00 to 0.00)		–0.02 (–0.05 to 0.02)	

Table 4a. Leg differences in ground reaction forces (16 RHA versus 9 THA) at flat walking at 1.11 m/s (4 km/h), at top walking incline (TWI), and at patients' top walking speed (TWS). Values are mean (SD), between-group differences (Δ), or adjusted between-group differences with CI (aD)

	Max. weight acceptance (N)		Max. push of force (N)		Mid stance support (N)		Impulse (N*s)	
	RHA	THA	RHA	THA	RHA	THA	RHA	THA
Walking 1.11 m/s								
O	842 (77)	814 (78)	817 (65)	801 (34)	648 (59)	639 (37)	444 (460)	441 (31)
CL	824 (98)	845 (45)	818 (86)	811 (37)	638 (73)	650 (46)	445 (60)	455 (29)
Δ	18 (84)	–31 (63)	–1 (65)	–11 (29)	10 (53)	–11 (57)	–1 (42)	–14 (38)
aΔ	–20 (–90 to 50)		–5 (–59 to 50)		–14 (–72 to 45)		–1 (–42 to 40)	
TWS								
O	1,132 (153)	977 (103)	751 (224)	748 (94)	368 (115)	479 (98)	338 (45)	325 (37)
CL	1,124 (114)	1,106 (85)	766 (176)	803 (139)	359 (109)	460 (83)	343 (30)	353 (25)
Δ	8 (100)	–129 (130)	–15 (67)	–56 (67)	10 (60)	20 (54)	–6 (25)	–28 (52)
aΔ	–141 (–261 to –20)		–48 (–120 to 23)		6 (–52 to 65)		–25 (–65 to 15)	
TWI								
O	812 (95)	769 (80)	840 (97)	827 (52)	596 (55)	635 (54)	435 (53)	433 (26)
Cl	826 (73)	799 (57)	881 (99)	841 (49)	599 (61)	621 (50)	454 (52)	451 (43)
Δ	–14 (65)	–29 (65)	–41 (50)	–13 (28)	–3 (39)	14 (53)	–19 (31)	–18 (27)
a Δ	–14 (–83 to 54)		29 (–17 to 75)		17 (–29 to 64)		–3 (–33 to 29)	

Table 4b. Sub-analysis of patients with 1 resurfacing (RHA) and 1 total hip arthroplasty (THA) (n = 5) and patients with a bilateral THA (n = 4). Values are mean (SD) or between-group differences with CI (D)

	Max. weight acceptance (N)		Max. push of force (N)		Mid stance support (N)		Impulse (N*s)	
**RHA + THA (n = 5)**	RHA	THA	RHA	THA	RHA	THA	RHA	THA
Walking 1.11 m/s	833 (47)	814 (41)	784 (27)	781 (33)	650 (23)	643 (43)	422 (26)	420 (26)
D	19 (–4 to 42)		3 (–36 to 43)		6 (–49 to 62)		2 (–14 to 17)	
TWS	1,075 (107)	1,030 (77)	726 (64)	732 (76)	464 (49)	486 (47)	336 (26)	336 (25)
D	45 (–63 to 153)		–6 (–80 to 68)		–22 (–65 to 20)		0 (–24 to 24)	
TWI	797 (32)	801 (33)	794 (47)	805 (28)	611 (46)	605 (60)	415 (22)	427 (18)
D	–4 (–57 to 50)		–11 (–71 to 50)		6 (–20 to 33)		–12 (–30 to 7)	
**THA + THA (n = 4)**	THA	THA	THA	THA	THA	THA	THA	THA
Walking 1.11 m/s	812 (110)	791 (123)	756 (103)	739 (113)	611 (77)	591 (77)	396 (72)	386 (76)
D	21 (–45 to 88)		18 (–36 to 72)		–20 (5 to 34)		9 (–4 to 23)	
TWS	1,075 (63)	1,072 (47)	752 (127)	749 (124)	487 (54)	492 (63)	352 (50)	348 (51)
D	2 (–82 to 87)		3 (–2 to 8)		–4 (–69 to 61)		3 (–24 to 31)	
TWI	867 (17)	832 (50)	810 (43)	789 (23)	593 (49)	615 (74)	416 (28)	415 (28)
D	35 (–17 to 90)		21 (–12 to 55)		–21 (–62 to 20)		1 (–12 to 13)	

Table 5. Hip range of motion differences at flat walking at 1.11 m/sec (4 km/h), at top walking incline (TWI), and at patients' top walking speed (TWS) in 16 RHA and 9 THA. Values are mean (SD), between-group differences (Δ), or adjusted between-group differences with CI (aD)

	RHA			THA			
	O	CL	Δ	O	CL	Δ	aΔ
Mean max. hip flexion							
Walking 1.11 m/s	35 (6)	37 (6)	–1.6 (4.8)	33 (8)	35 (5)	–2.1 (5.8)	1.2 (–3 to 5.4)
TWS	47 (5)	47 (6)	0.3 (3.9)	38 (6)	42 (6)	–3.5 (5.1)	–2.8 (–7.4 to 1.8)
TWI	55 (6)	57 (7)	–2.1 (5.0)	56 (13)	53 (6)	3.0 (10.5)	6.6 (–1.0 to 14)
Mean max. hip extension							
Walking 1.11 m/s	3 (7)	1 (7)	1,7 (5.4)	1 (7)	1 (6)	0.1 (5.9)	–1.9 (–7.8 to 4)
TWS	1 (6)	–1 (6)	1.6 (5.7)	1 (9)	0 (6)	1.4 (7.2)	–0.9 (–7.6 to 5.8)
TWI	4 (8)	1 (8)	2.9 (4.6)	4 (8)	5 (6)	–1.3 (7.7)	–5.5 (–11.7 to 0.6)

Table 6. Sub-analysis of patients with bilateral hip implants: 1 resurfacing (RHA) and 1 total hip arthroplasty (THA) (n = 5) and patients with a bilateral THA (n = 4). Values are mean (SD) and between-group differences with CIs (Δ)

	Spatiotemporal parameters				Kinematic parameters			
	Mean stride length (m)		Mean stride time (s)		Mean max. hip flexion		Mean max. hip extension	
RHA + THA (n = 5)	RHA	THA	RHA	THA	RHA	THA	RHA	THA
Walking 1.11 m/s	1.18 (0.05)	1.18 (0.05)	1.06 (0.05)	1.06 (0.05)	33 (5)	34 (5)	4 (5)	3 (4)
Δ	0.00 (–0.00 to 0.00)		0.00 (–0.00 to 0.00)		–1 (–6 to 3)		2 (–3 to 6)	
TWS	1.64 (0.18)	1.64 (0.18)	0.87 (0.07)	0.87 (0.07)	41 (7)	42 (6)	6 (5)	6 (5)
Δ	0.00 (–0.00 to 0.00)		0.00 (–0.00 to 0.00)		–1 (–6 to 3)		0 (–6 to 6)	
TWI	1.18 (0.07)	1.18 (0.07)	1.07 (0.04)	1.07 (0.04)	52 (7)	54 (6)	3 (4)	0 (5)
Δ	0.00 (0.00 to 0.00)		0.00 (–0.00 to 0.00)		–2 (–7 to 4)		3 (–2 to 8)	
THA + THA (n = 4)	THA	THA	THA	THA	THA	THA	THA	THA
Walking 1.11 m/s	1.18 (0.09)	1.18 (0.09)	1.06 (0.09)	1.06 (0.09)	35 (4)	34 (6)	1 (3)	1 (5)
Δ	0.00 (–0.00 to 0.00)		0.00 (–0.00 to 0.00)		1 (–3 to 4)		0 (–7 to 7)	
TWS	1.57 (0.11)	1.57 (0.11)	0.87 (0.12)	0.87 (0.12)	48 (16)	42 (8)	4 (3)	2 (9)
Δ	0.00 (–0.00 to 0.00)		0.00 (–0.00 to 0.00)		6 (–19 to 31)		2 (–11 to 14)	
TWI	1.16 (0.09)	1.16 (0.09)	1.05 (0.08)	1.05 (0.09)	54 (8)	55 (7)	–1 (4)	0 (6)
Δ	0.00 (–0.00 to 0.00)		0.00 (–0.00 to 0.00)		–1 (–7 to 5)		–1 (–6 to 5)	
